# Selective attention decoding in bimodal cochlear implant users

**DOI:** 10.3389/fnins.2022.1057605

**Published:** 2023-01-11

**Authors:** Hanna Dolhopiatenko, Waldo Nogueira

**Affiliations:** Department of Otolaryngology, Hannover Medical School and Cluster of Excellence Hearing4all, Hanover, Germany

**Keywords:** cochlear implant, selective attention, electric acoustic stimulation, electrophysiological measures, central integration, bimodal hearing, bimodal stimulation, electroencephalography

## Abstract

The growing group of cochlear implant (CI) users includes subjects with preserved acoustic hearing on the opposite side to the CI. The use of both listening sides results in improved speech perception in comparison to listening with one side alone. However, large variability in the measured benefit is observed. It is possible that this variability is associated with the integration of speech across electric and acoustic stimulation modalities. However, there is a lack of established methods to assess speech integration between electric and acoustic stimulation and consequently to adequately program the devices. Moreover, existing methods do not provide information about the underlying physiological mechanisms of this integration or are based on simple stimuli that are difficult to relate to speech integration. Electroencephalography (EEG) to continuous speech is promising as an objective measure of speech perception, however, its application in CIs is challenging because it is influenced by the electrical artifact introduced by these devices. For this reason, the main goal of this work is to investigate a possible electrophysiological measure of speech integration between electric and acoustic stimulation in bimodal CI users. For this purpose, a selective attention decoding paradigm has been designed and validated in bimodal CI users. The current study included behavioral and electrophysiological measures. The behavioral measure consisted of a speech understanding test, where subjects repeated words to a target speaker in the presence of a competing voice listening with the CI side (CIS) only, with the acoustic side (AS) only or with both listening sides (CIS+AS). Electrophysiological measures included cortical auditory evoked potentials (CAEPs) and selective attention decoding through EEG. CAEPs were recorded to broadband stimuli to confirm the feasibility to record cortical responses with CIS only, AS only, and CIS+AS listening modes. In the selective attention decoding paradigm a co-located target and a competing speech stream were presented to the subjects using the three listening modes (CIS only, AS only, and CIS+AS). The main hypothesis of the current study is that selective attention can be decoded in CI users despite the presence of CI electrical artifact. If selective attention decoding improves combining electric and acoustic stimulation with respect to electric stimulation alone, the hypothesis can be confirmed. No significant difference in behavioral speech understanding performance when listening with CIS+AS and AS only was found, mainly due to the ceiling effect observed with these two listening modes. The main finding of the current study is the possibility to decode selective attention in CI users even if continuous artifact is present. Moreover, an amplitude reduction of the forward transfer response function (TRF) of selective attention decoding was observed when listening with CIS+AS compared to AS only. Further studies to validate selective attention decoding as an electrophysiological measure of electric acoustic speech integration are required.

## 1. Introduction

The growing group of cochlear implant (CI) users includes subjects with preserved acoustic hearing on the opposite side to the CI. The combination of electric and contralateral acoustic stimulation, also referred to as bimodal stimulation, usually results in an improvement in sound localization (Ching et al., 2004; Potts et al., 2009; Arndt et al., 2011; Firszt et al., 2012; Prejban et al., 2018; Galvin et al., 2019), music perception (Kong et al., 2005; Ching et al., 2007), tinnitus suppression (Van de Heyning et al., 2008; Galvin et al., 2019) and quality of life (Galvin et al., 2019) compared to monaural listening. Moreover, subjects with bimodal stimulation can integrate electric and acoustic information to improve their speech understanding (Ching et al., 2004; Kong et al., 2005; Dorman et al., 2008; Potts et al., 2009; Vermeire and Van de Heyning, 2009; Yoon et al., 2015; Devocht et al., 2017). However, the observed benefits present high variability across subjects (Ching et al., 2007; Crew et al., 2015) and some subjects even experience worsened speech performance with bimodal stimulation (Litovsky et al., 2006; Mok et al., 2006; Galvin et al., 2019). This variability in speech outcomes with bimodal listening may be associated with the effectiveness of the speech integration between electric and acoustic stimulation. Some previous works suggested that this integration has a central origin (Yang and Zeng, 2013; Reiss et al., 2014; Fowler et al., 2016; Balkenhol et al., 2020). However, the integration mechanisms and its impact on bimodal benefit requires investigation.

Different mechanisms might contribute into electric acoustic integration of speech: the integration of complementary speech information conveyed through electric and acoustic stimulation, the integration of similar speech information conveyed electrically and acoustically or the combination of the two mechanisms. Reiss et al. (2014) showed that bimodal CI users obtain abnormal spectral integration, which might lead to speech perception interference between the electric and the acoustic stimulation sides. To solve this, they suggested to reduce overlap in frequency information transmitted through electric and acoustic stimulation (Reiss et al., 2012a,b). Fowler et al. (2016) also investigated the reduction of frequency overlap in bimodal CI users and observed that subjects with better residual hearing (< 60 dB HL at 250 and 500 Hz) might benefit when low frequency information is removed on the CI side. In contrast, Fu et al. (2017) showed that bimodal perception is not significantly impacted when changing the CI input low-cutoff frequency, claiming that bimodal CI users do not benefit from the mismatch correction. However, that study was conducted using a vocoder to simulate bimodal hearing in normal hearing subjects. The study of Kong and Braida (2011) assumed that bimodal listeners do not integrate available cues from both listening sides but rather rely on the cues processed by the dominant stimulation. However, Yoon et al. (2015) demonstrated that bimodal benefit does not depend on the performance of the dominant acoustic side alone but can be predicted by the difference between performances of the two stimulation modalities. Therefore, authors concluded that the bimodal benefit is a result of the integration between electric and acoustic stimulation.

The benefit of electric acoustic stimulation in bimodal CI users is usually measured behaviorally using clinical speech performance tests. These tests suffer from test-retest variability, cannot be applied to people with missing behavioral response and do not provide insights about the underlying physiological mechanisms related to electric acoustic integration. The understanding of these physiological mechanisms may provide novel approaches to program the CI and consequently improve speech perception in bimodal CI users. EEG is promising as an objective measure of speech integration for bimodal listening, however, its application is challenging because it is influenced by the CI electrical artifact.

Nowadays, there is a growing interest in the use of cortical auditory evoked potentials (CAEPs) as an objective measure of sound perception in NH listeners (Martin et al., 2007; Stapells, 2009; Papesh et al., 2015) and in CI users (Pelizzone et al., 1987; Ponton et al., 1996; Firszt et al., 2002; Maurer et al., 2002; Sharma et al., 2002). It has been shown that CAEPs provide information about binaural interaction at central level in NH listeners by analyzing the deviation of binaural responses from the sum of monaural responses (i.e. binaural interaction component (BIC) analysis) (McPherson and Starr, 1993; Jancke et al., 2002; Henkin et al., 2015). CAEPs were also measured in people with asymmetric hearing loss, revealing that the sound at cortical level is processed similarly for acoustic alone and electric alone stimulation (Sasaki et al., 2009; Balkenhol et al., 2020; Wedekind et al., 2020, 2021). However, the amount of studies investigating electric acoustic integration at cortical level in bimodal CI users is limited. The current study investigates the possibility to record CAEPs when listening with the CI side (CIS) alone, the acoustic side (AS) alone and both sides simultaneously (CIS+AS).

One of the main disadvantages of CAEPs is that they require the use of relatively short and simple stimuli. Therefore, the relation between CAEPs and speech understanding is not easy to establish. Another alternative EEG measure, which recently has gained significant interest as an objective measure is neural tracking of the envelope of an attended speech source (Ding and Simon, 2012; Giraud and Poeppel, 2012; Power et al., 2012; Mirkovic et al., 2015; OSullivan et al., 2015). The paradigm in which in addition to the attended speaker also an ignored speaker is introduced, is called selective attention decoding. Two linear approaches exist for selective attention decoding, the forward and the backward models. Both approaches are based on least mean square error minimization between audio features and neural signals. Most previous studies performed stimulus-response mapping in the forward direction, i.e. using forward models to investigate how the system generates or encodes information (Haufe et al., 2014). By applying the forward model, the temporal response function (TRF), which describes the relationship between speech and neural recordings, is obtained. The morphology of the TRF resembles the classical N1P2 complex of the late evoked potentials (Lalor et al., 2009; Crosse et al., 2016). Analysis of the N1P2 TRF complex might provide different information than the N1P2 complex of CAEPs due to the utilization of more ecological speech stimuli, as selective attention is decoded using continuous speech streams. In order to investigate how speech features are decoded from the neural representation, one can apply the backward model (Mesgarani et al., 2009; Ding and Simon, 2012; Pasley et al., 2012; Mirkovic et al., 2015; OSullivan et al., 2015; Crosse et al., 2016). By using the backward model, the speech stimulus is reconstructed from the neural activity recordings. The backward model explores the accuracy of decoding by analyzing speech features of reconstructed and original speech stimuli.

Recently, the possibility to predict speech intelligibility from selective attention decoding has been shown in NH listeners (Keitel et al., 2018; Vanthornhout et al., 2018; Dimitrijevic et al., 2019; Etard and Reichenbach, 2019; Lesenfants et al., 2019), in hearing impaired listeners with hearing aids (Petersen et al., 2017) and in bilateral (Paul et al., 2020) and monaural CI users (Nogueira and Dolhopiatenko, 2022). However, the application of such objective measures in CI users is still challenging because of the CI electrical artifact leaking into the EEG recordings (Hofmann and Wouters, 2010; Somers et al., 2010; Deprez et al., 2017). Artifact rejection techniques such as independent component analysis (ICA) can suppress artifacts in EEG, however, the full removal of the CI electrical artifact can not be ensured. Nevertheless, some previous works showed that it is still feasible to decode selective attention in CI users (Nogueira et al., 2019a,b; Aldag et al., 2022). In this regard, these previous works showed that the maximum differentiation between the attended and the ignored speaker occurs at 200–400 ms after stimulus onset showing a minimization effect of the CI electrical artifact at this time interval. However, as selective attention is recorded to the continuous speech, the impact of the CI electrical artifact cannot be fully discarded and more evidences that selective attention decoding is possible in CI users are necessary. Therefore, the main goal of the current study is to confirm the feasibility to decode selective attention in bimodal CI users, which will provide further evidence on the use of continuous EEG recordings to speech stimuli in this population, despite the presence of the CI electrical artifact. This work investigates selective attention decoding in bimodal CI users when listening with CIS only, AS only and both sides together (CIS+AS). It is hypothesized that combined electric and acoustic stimulation results in improved selective attention decoding with respect to listening with CIS alone. If this hypothesis is confirmed, it can be concluded that it is possible to decode selective attention in CI users, as the additional neural activity provided by the acoustic stimulation is used to improve the decoding, even if CI artifact is present. Moreover, the confirmation of the main hypothesis might open the possibility to further investigate selective attention decoding as a measure of speech integration between electric and acoustic stimulation in bimodal CI users using continuous speech which is a natural and ecologically valid signal.

To find a descriptive link between speech understanding and selective attention decoding, the current study also included a behavioral measure. The behavioral measure consisted of a speech understanding performance test to a target speaker in the presence of a competing talker. Speech material was presented using the three listening modes (CIS only, AS only, and CIS+AS). The second part of the study included recording of EEG. The possibility to record cortical responses to short stimuli with all three listening modes was demonstrated through CAEPs. Afterwards, selective attention decoding, which is a novel approach when applied to bimodal CI users, was measured. In the selective attention paradigm, a target and a competing talker were presented to the subjects using the three listening modes (CIS only, AS only, CIS+AS). The main goal of this study is to investigate the feasibility to decode selective attention in CI users despite the presence of CI electrical artifact. Furthermore, first attempts to investigate the potential of selective attention decoding as a speech integration measure between electric and acoustic stimulation in bimodal CI users were conducted.

## 2. Materials and methods

### 2.1. Participants

Ten subjects participated in the study (mean age: 57.7). All participants were implanted with an Oticon Medical CI and had a device experience of 6–48 months. Demographics of the participants are shown in Table 1. Prior to the experiment, all participants provided written informed consent and the study was carried out in accordance with the Declaration of Helsinki principles, approved by the Ethics Committee of the Hannover Medical School.

**Table 1:** Demographics of participants.

**ID**	**Sex**	**Age**	**Hearing aid**	**Implanted side**	**Duration of deafness (y)**	**CI Experience (y)**	**Stream to be attended**
1	M	63	Yes	Left	55	4	Male
2	F	62	No	Right	3	4	Female
3	F	54	No	Left	42	4	Male
4	M	62	Yes	Left	13	1.5	Male
5	M	68	No	Left	4	2	Female
6	M	47	no	Left	1	3	Female
7	M	73	No	Right	12	4	Male
8	M	57	Yes	Right	19	0.5	Female
9	M	30	No	Left	0.07	3.4	Male
10	F	61	No	Right	15	1.5	Female

**Figure 1:**
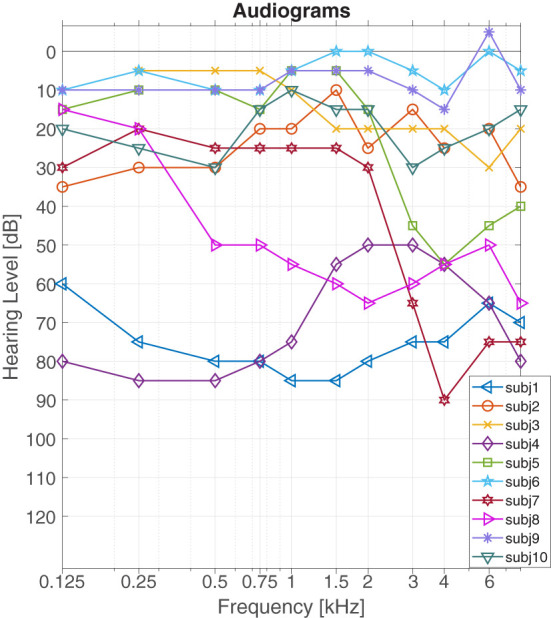
Audiograms of study participants.

A pure tone audiogram on the non-implanted ear in unaided condition was measured *via* a calibrated audiometry system (CAS AD2117, Audio-DATA, Duvensee, Germany). The audiograms for the study participants are presented in Figure 1.

### 2.2. Behavioral paradigm

The German Hochmair-Schulz-Moser sentence test (HSM test) (Hochmair-Desoyer et al., 1997) was used to assess speech understanding behaviorally. Each list consists of 20 semantically structured sentences, uttered by a male or by a female talker. Two sentence lists were presented to the acoustic side only (AS only), to the CI side only (CIS only) and to both sides simultaneously (CIS+AS). Target and interference speech streams were co-located and presented at 0 dB signal-to-interference ratio (SIR) between the target (male/female) and the interference (female/male) speech stream. Subjects were instructed to attend to the target talker and to repeat all words after each sentence. The speech stream to be attended was randomized within subjects and is indicated in Table 1. The attended speech stream was kept the same through the whole experiment. The speech understanding performance score was calculated in percentage of correct recalled words per listening mode. Speech material was presented to the CI side *via* the Oticon Bluetooth Streamer and to the acoustic side *via* inner-ear phones (E-A-RTONE Gold 3A, 3M, St. Paul, Minneapolis). For subjects wearing a hearing aid on the contralateral side to the CI, speech material presented to the AS was preprocessed using a digital hearing aid implemented in a PC. The hearing aid was based on the half-gain rule amplification according to the measured audiogram (Lybarger, 1963). This hearing aid implementation has been successfully used in previous studies in our group (Krüger et al., 2022). Stimulus presentation was controlled by the Presentation Software (Neurobehavioral Systems, Inc., Berkeley, CA, United States; version 20.1). The presentation level for the CIS and AS was set to a most comfortable level, using a seven point loudness rating-scale (from 1 to 7: from extremely soft to extremely loud; 4 - most comfortable level).

### 2.3. Electrophysiological paradigm

The electrophysiological part of the experiment consisted of EEG recordings. The recording was conducted in an electromagnetically and acoustically shielded booth. High-density continuous EEG was recorded using a SynAmps RT System with 64 electrodes mounted in a customized, infracerebral electrode cap (Compumedics Neuroscan, Australia). The reference electrode was placed on the nose tip; two additional electrodes were placed on the mastoids. Impedances were controlled and maintained below 15 kΩ. Electrodes with high impedance were excluded from further analysis. Each subject was instructed to sit relaxed, avoid any movements, and to keep their eyes open in order to minimize physiological artifacts. All material was presented *via* Bluetooth Streaming to the CIS and *via* inner-ear phones to the AS. For AS in CI users with a hearing aid on the contralateral side, all presented material was processed with the same digital hearing aid and adjusted in loudness to their MCL exactly in the same manner as in the behavioral paradigm described in Section 2.2.

#### 2.3.1. Cortical auditory evoked potentials

##### 2.3.1.1. Stimuli

To maximize responses, a broadband noise of 50 ms duration was used as a stimulus. To ensure time synchronization between both listening sides, the delay between electric and acoustic stimulation needs to be considered. The technical CI delay was measured using an oscilloscope and a research implant unit. The stimulus was presented through the Bluetooth Streamer to the CI processor, and the delay between audio start and the start of electrical stimulation resulted in 30 ms. On the acoustic side however, the delay between stimulus onset and the auditory nerve response is frequency dependent and variable across subjects (Elberling et al., 2007). It was estimated from the literature that the average delay for a stimulus to travel from the outer ear to the auditory nerve is 7 ms (Elberling et al., 2007), therefore, the stimulus for AS started 23 ms after the onset of the stimulus on the CIS. Note that delay compensation was conducted at group level and not adjusted individually for each subject. The stimuli were presented with an inter-stimulus interval of 1 s. In total, 100 trials were recorded. The EEG data was recorded with a sampling rate of 20 kHz.

##### 2.3.1.2. Processing

Recorded EEG data was processed through the EEGLAB MATLAB toolbox (Delorme and Makeig, 2004). ICA based on second-order blind identification (SOBI) was applied to the recorded data to remove physiological and CI electrical artifacts (Kaur and Singh, 2015). Data recorded with CIS only and with CIS+AS listening mode was concatenated prior to SOBI in order to ensure equal portion of the removed CI electrical artifact in both listening modes. The topology and the signal in the time and spectral domain were visually analyzed for each component. On average 1.8 (std:±0.64) components for each subject were removed from the data. EEG with the suppressed artifacts was afterwards epoched in the time interval ranging from –200 to 1,000 ms. The CAEPs were obtained from the vertex electrode (Cz). The signal was filtered between 1 and 15 Hz and re-referenced to the mean of the two mastoid electrodes.

#### 2.3.2. Selective attention decoding

##### 2.3.2.1. Stimuli

For the selective attention paradigm HSM sentence lists with a male and a female talker at 0 dB SIR were used. The speech stream to be attended was kept the same as in the behavioral part of the experiment (Table 1). For each listening condition (CIS only, AS only, and CIS+AS) 8 lists were presented, resulting in approximately 6 min of stimulation per listening mode. To extend the training dataset for the decoder, additional speech material consisting of two audio story books were used. The story books included two German narrations (“A drama in the air” by Jules Verne, narrated by a male speaker and “Two brothers” by the Grimm brothers, narrated by a female speaker) at 0 dB SIR. In total, 36 min of story (12 min per listening mode) were presented. To ensure the continuous engagement of the CI user when listening to the corresponding speaker, questions to the context of the presented speech material were asked every 2 min. The presented speech material was randomized across listening conditions to avoid the influence of the material. EEG data was recorded with a sampling rate of 1,000 Hz.

##### 2.3.2.2. Processing

EEG data was processed offline in MATLAB (MATLAB, 2018) and the EEGLAB toolbox (Delorme and Makeig, 2004). SOBI artifact rejection was applied to the EEG data to suppress physiological and CI electrical artifact. The location of the CI and the signal in the time and spectral domain of each component were analyzed. On average, 3.5 (std: ±1.08) components were removed from the data. Afterwards, the EEG data was split into the trials corresponding with the duration of each sentence list and 1 min segments of the story. Next, the digital signal was band-pass filtered for frequencies 2–8 Hz and downsampled to 64 Hz. The envelopes of the original attended and unattended speech streams were extracted through the Hilbert transform. The envelopes were filtered with a low-pass filter having cut-off frequency of 8 Hz and downsampled to 64 Hz. Selective attention was analyzed using the forward and the backward model approaches (Crosse et al., 2016). By applying the forward model, the TRF was obtained. By using the backward model the speech stimulus was reconstructed from the neural activity recordings. The correlation coefficient between the original envelope of the attended audio and the reconstructed envelope (attended correlation coefficient ρ_*A*_) as well as the correlation coefficient between the original envelope of the unattended audio and the reconstructed envelope (unattended correlation coefficient ρ_*U*_) were calculated. Selective attention decoding was analyzed in terms of ρ_*A*_ and the difference between ρ_*A*_ and ρ_*U*_ (ρ_*Diff*_). Both, forward and backward models were applied across time lag. The time lag performs a time shift of the EEG signal that reproduces the physiological delay between the audio presentation and its processing up to the cortex (OSullivan et al., 2015). In total 38 lags spanning the interval from 16 to 608 ms were used. The lag window, over which reconstruction was conducted, was set to △ = 16 ms. The regularization parameter λ was set to 100 to maximize the peak amplitudes of the TRF for the forward model and to 0.01 to maximize the difference between the attended and unattended correlation coefficients for the backward model. Further details on the analysis of TRFs and correlation coefficients across λ can be found in the Supplementary material. For more details on the reconstruction procedure see Nogueira et al. (2019a,b). A classical leave-one-out cross-validation approach was used to train and test the decoder. HSM lists and the story were used to train the decoder. Only HSM sentences were used for testing, resulting in 8 folds for cross-validation (corresponding to the amount of lists) with each listening mode.

### 2.4. Statistical analysis

Statistical analysis was carried out with the SPSS software (version 26, IBM). The effect of listening mode on the investigated parameters was explored through a repeated measures analysis of variance (ANOVA). Pairwise comparisons between listening modes were conducted with *post-hoc* analysis based on the *t*-test for each pair of observations. To avoid type I error for multiple comparisons, Bonferroni correction was applied. For non-normally distributed data, the non-parametric Friedman test followed by a *post-hoc* Wilcoxon signed-rank test for pairwise comparisons was applied to the data.

## 3. Results

### 3.1. Behavioral paradigm

**Figure 2:**
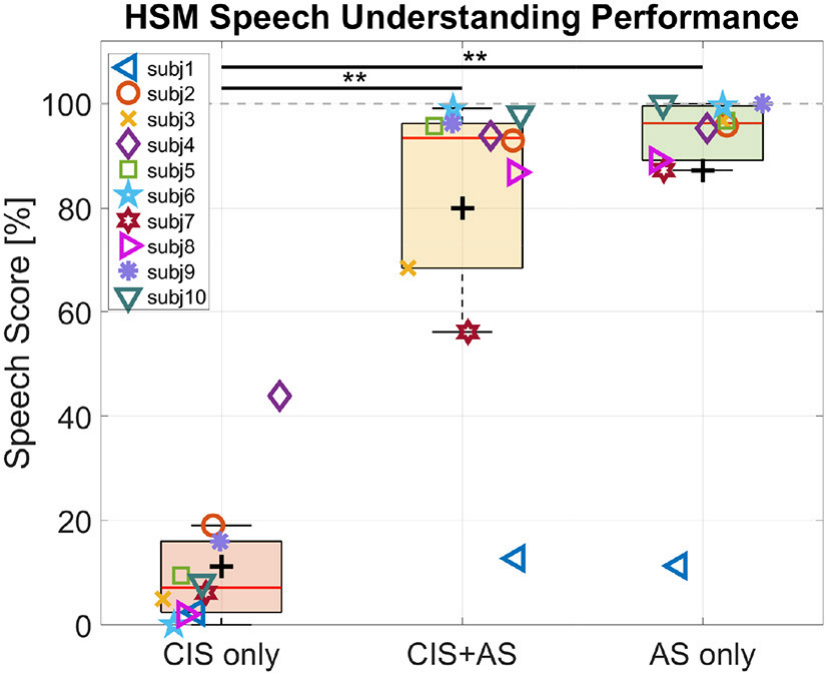
Behavioral speech understanding performance for three listening modes: CIS only, CIS+AS, and AS only. Asterisk indicates significant difference between a pair of conditions revealed through the Wilcoxon signed-rank test.

Figure 2 shows the individual speech understanding performance for each listening mode.

On average, the highest score was observed with AS only listening mode (87.18%), followed by CIS+AS listening mode (80.01%). The lowest score was obtained with CIS only (11.21%) and can be explained by the high difficulty of the task for the participants when listening with CIS alone. A Friedman test revealed a significant effect of listening mode on speech performance scores $\left[\chi_{\left(2\right)}^{2}=18.200;p<0.001\right]$. *Post-hoc* analysis with the Wilcoxon signed-rank test with a Bonferroni correction resulted in a significant effect for the pairs CIS+AS—CIS only (*p* = 0.005) and AS only—CIS only (*p* = 0.005). No significant difference between CIS+AS and AS only was observed.

### 3.2. Cortical auditory evoked potentials

**Figure 3:**
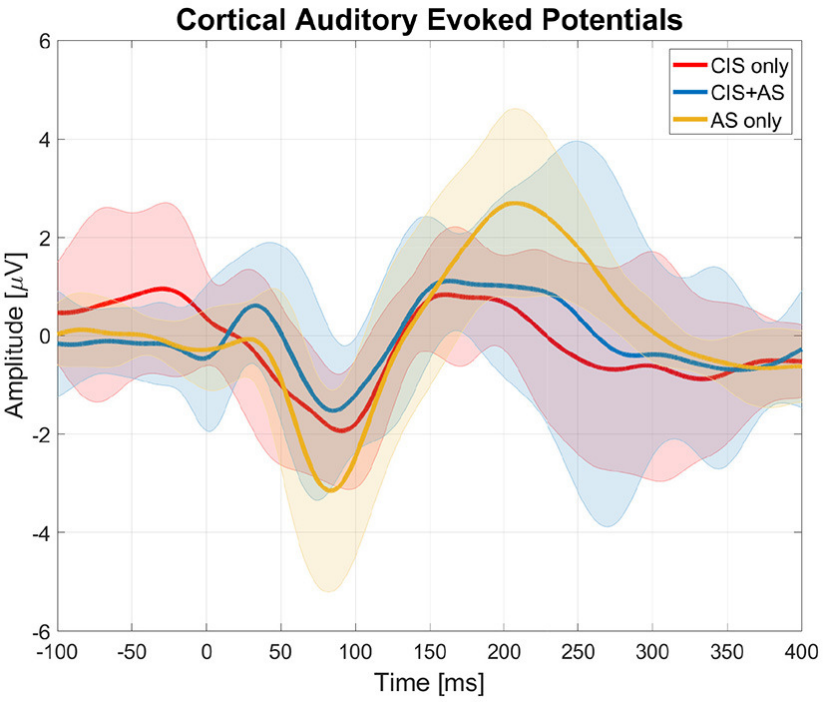
Cortical auditory evoked potentials (CAEPs) obtained from the central electrode (Cz) with three listening modes (CIS only, AS only, and CIS+AS) averaged across subjects. The thick lines represent the mean values across subjects and the shaded areas represent the standard deviation across subjects.

Figure 3 presents the averaged CAEPs across subjects after SOBI artifact rejection for the three listening modes (CIS only, AS only, and CIS+AS). Subject 1 was excluded from the analysis due to the low quality of the recorded signal.

In general, it was possible to distinguish the cortical response with all three listening modes. The peak-to-peak amplitude of the N1P2 was estimated for each subject individually. An ANOVA analysis revealed a significant effect of listening mode on the N1P2 amplitude [*F*_(2, 16)_ = 6.544;*p* = 0.008]. A *post-hoc t*-test with Bonferroni correction revealed a significantly higher N1P2 amplitude with AS only than with CIS only listening mode (*p* = 0.014). No significant differences between CAEPs recorded with CIS only and CIS+AS listening modes were found.

### 3.3. Selective attention decoding

#### 3.3.1. Temporal response function

**Figure 4:**
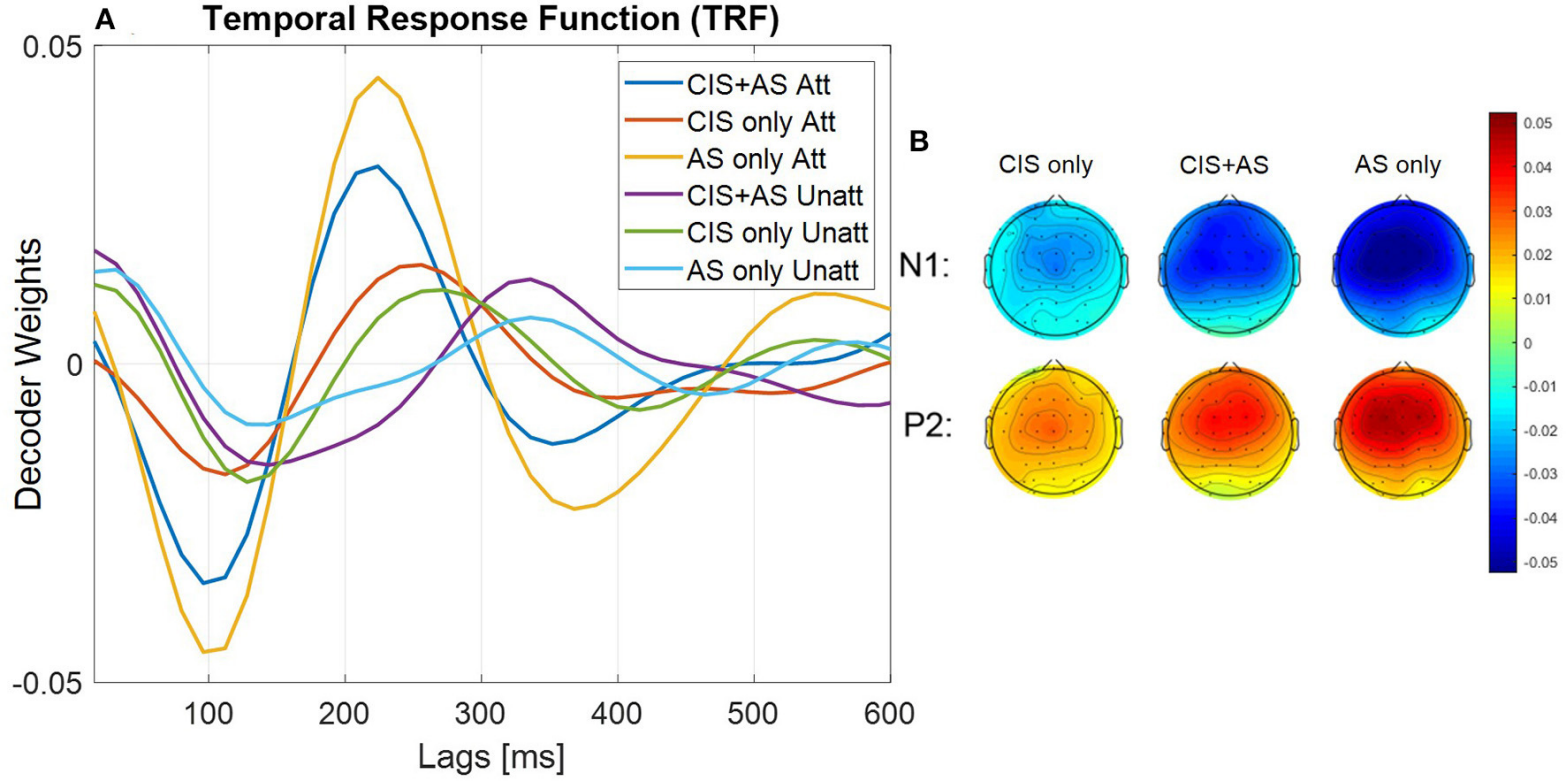
**(A)** Forward transfer response function (TRF) averaged across subjects. Attended TRF and unattended TRF are estimated using attended and unattended decoder respectively; **(B)** Topographical maps show TRF weights across subjects at first and second peaks of attended curve for each listening mode.

Figure 4A presents the mean TRF across subjects, where the TRF represents the decoder weights of the forward model approach. The TRF were analyzed comparing the first negative (N1) and second positive (P2) peaks for each listening mode and listener. The analysis revealed highest N1P2 peak-to-peak amplitude for the AS only, followed by the CIS+AS and the lowest amplitude for the CIS only listening mode. Moreover, weights of the TRF at the N1 and P2 peaks were estimated for each subject and presented in the form of topographical maps per each listening mode (Figure 4B). The weight distribution is similar across all listening modes, however, the activation power with the CIS only listening mode is visibly weaker.

**Figure 5:**
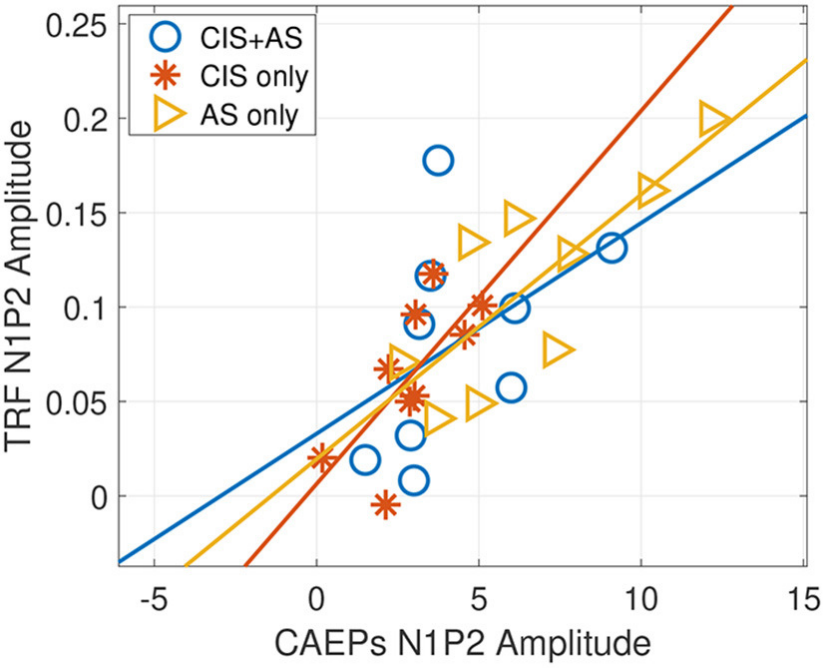
Pearson correlation between N1P2 peak amplitudes of the temporal response function (TRF) and N1P2 peak amplitudes of cortical auditory evoked potentials (CAEPs) with three listening modes: CIS only, CIS+AS, and AS only.

From Figure 4A, it can be observed that the latencies of the TRF peaks for the CIS+AS and the AS only listening modes are similar, while the latency for CIS only is delayed. Moreover, the amplitude of the N1P2 peak of the TRF was compared to the amplitude of the N1P2 peaks obtained from CAEPs presented in Section 3.2. A significant correlation between the N1P2 peak-to-peak amplitude from TRFs and CAEPs was observed for CIS only and AS only listening modes (CIS only: *r* = 0.715, *p* = 0.031; AS only: *r* = 0.793, *p* = 0.011) (Figure 5). For the CIS+AS listening mode, no significant correlation between the N1P2 amplitude derived from CAEPs and TRFs was found. This may be explained by the temporal delay correction between electric and acoustic stimulation implemented in CAEP measurements, which was not applied during the selective attention decoding experiment.

#### 3.3.2. Selective attention correlation coefficients

**Figure 6:**
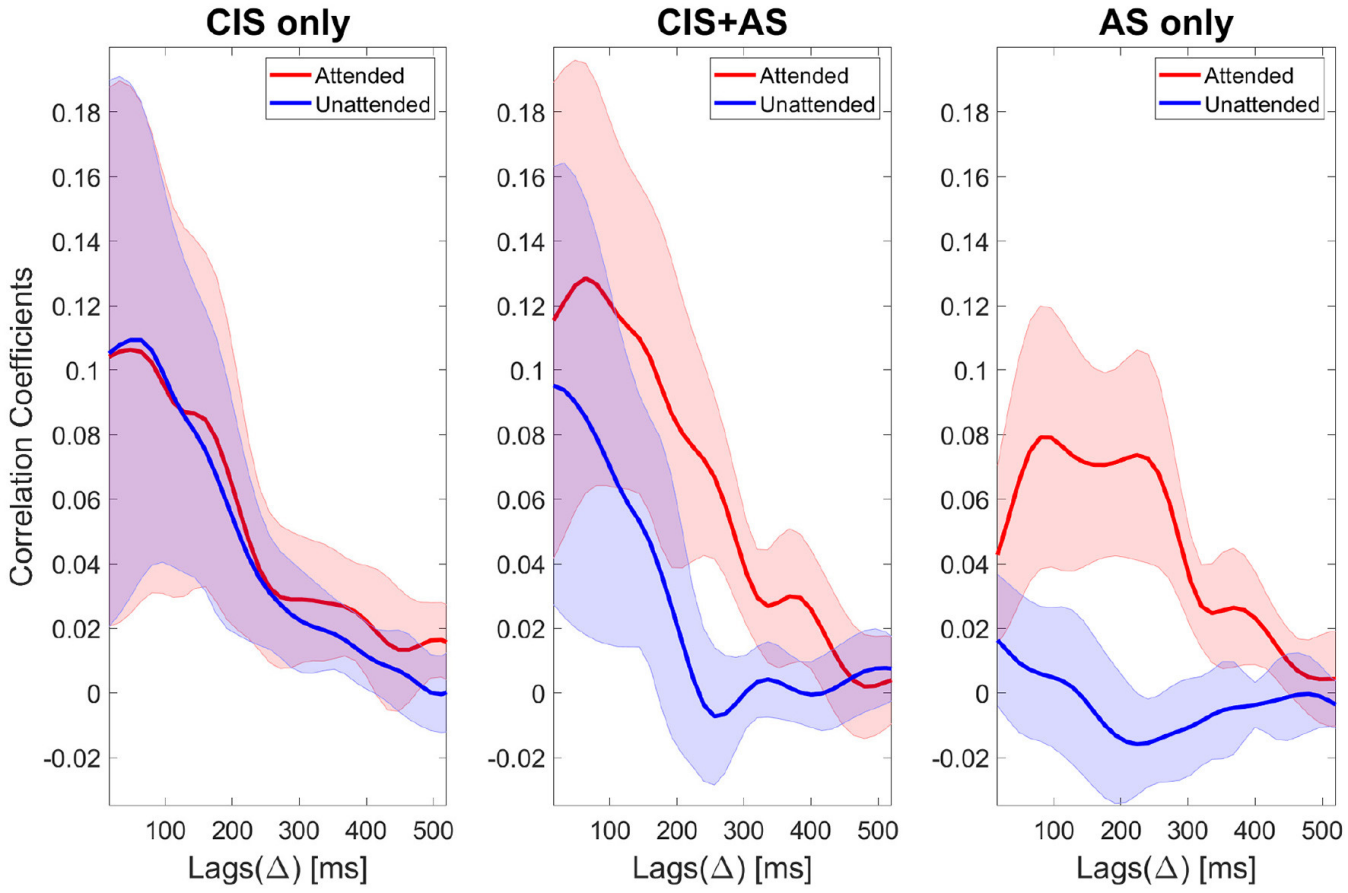
Attended correlation coefficients (red color) and unattended correlation coefficients (blue color) of selective attention decoding for three listening modes: CIS only **(left)**, CIS+AS **(center)**, and AS only **(right)**. Correlation coefficients are denoted as corr coeff and calculated across lags (△). The thick lines represent the mean values and the shaded areas represent the standard deviation across subjects.

Figure 6 presents the ρ_*A*_ and ρ_*U*_ coefficients across lags obtained from selective attention decoding using the backward model after SOBI artifact rejection. Note, that lag △ is used to time shift the EEG, modeling the physiological delay required for a sound to travel along the auditory pathway up to the cortex.

The correlation coefficients across lags for the AS only condition present a morphology consistent with the morphology reported in NH listeners (Nogueira et al., 2019a). In NH listeners, the typical morphology of the ρ_*A*_ curve presents two peaks at around 100 and 250 ms associated with different stages of neural processing. The correlation coefficients obtained with the CIS only and CIS+AS listening modes at early lags were higher than with AS only indicating a contribution of the CI electrical artifact. SOBI artifact rejection suppressed part of this artifact, however, full removal could not be achieved. In order to minimize the effect of the CI artifact, a later lag interval was chosen for further analysis. Based on previous works (Nogueira et al., 2019a,b), the chosen lag interval spanned the time between 208 and 304 ms, which also corresponds to the second peak of the ρ_*A*_ curve for the AS only condition (Figure 6). At that chosen lag interval, a *t*-test comparing the ρ_*A*_ and the ρ_*U*_ coefficients for each listening mode revealed a significant difference for the CIS+AS (*p* < 0.001) and for the AS only (*p* < 0.001) listening modes, but not for the CIS only mode (*p* = 0.405) due to the small differences between the ρ_*A*_ and the ρ_*U*_. This result confirms the possibility to decode selective attention in CI users despite the presence of CI electrical artifact.

**Figure 7:**
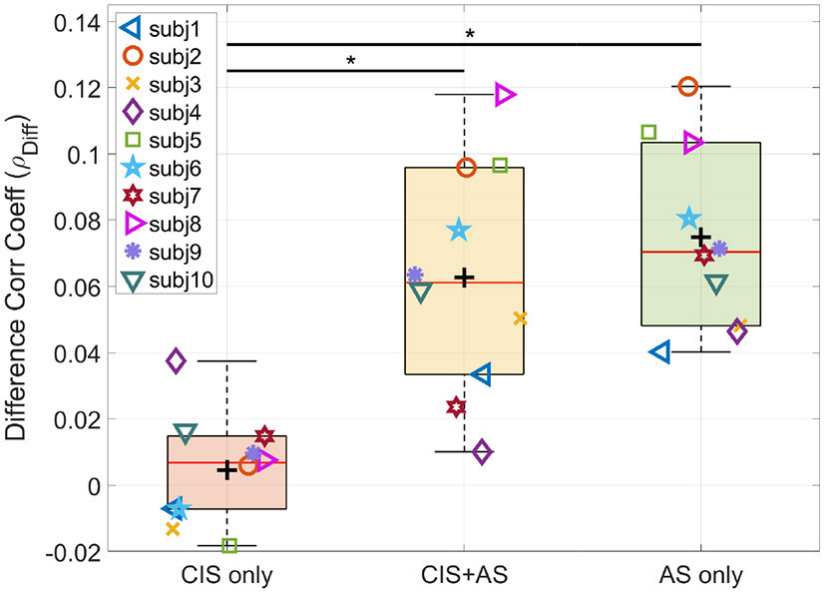
Difference between attended and unattended correlation coefficients (ρ_*Diff*_) of selective attention decoding at the 208–304 ms lag interval. Asterisk indicates significance between pair of observations revealed by the *t*-test.

Furthermore, we focus our analysis only on the difference between the attended and the unattended correlation coefficients (ρ_*Diff*_), which reduces the impact of the CI artifact (Nogueira and Dolhopiatenko, 2022). Figure 7 shows the ρ_*Diff*_ at the lag interval 208–304 ms for each listening mode. The ANOVA test revealed a significant effect of listening mode on the ρ_*Diff*_ *F*_(2, 18)_ = 23.640;*p* < 0.001. The *post-hoc* pairwise *t*-test comparison showed a significant difference for the pairs CIS+AS—CIS only (*p* = 0.003) and AS only—CIS only (*p* = 0.011). Note that the behavioral speech understanding scores were also significantly different for the same pairs of comparisons.

**Figure 8:**
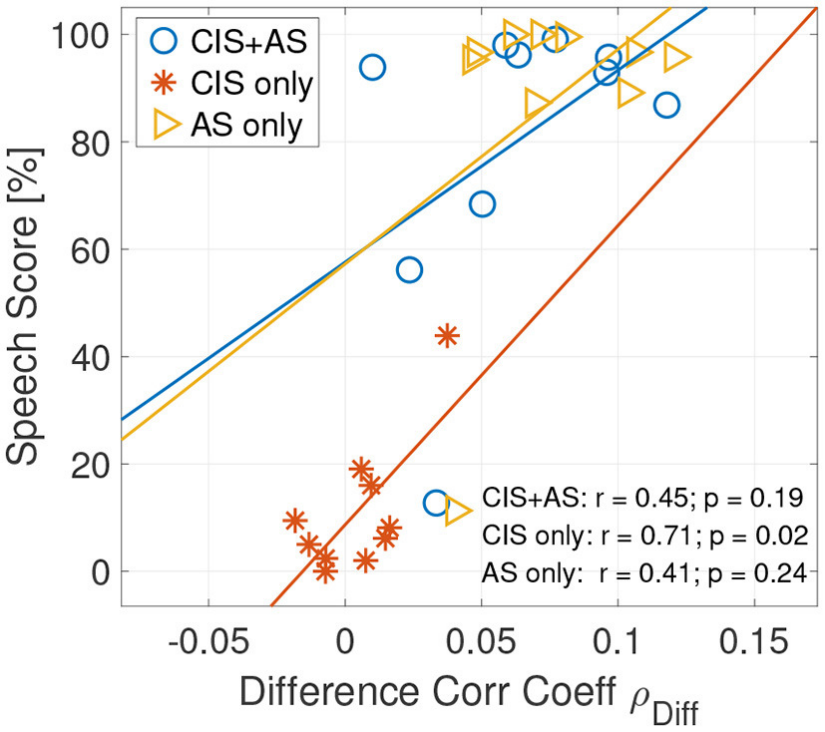
Correlation plot between the speech understanding scores and the attended correlation coefficients ρ_*A*_ **(left)** and between the speech understanding scores and the difference between the attended and the unattended correlation coefficients ρ_*Diff*_ **(right)** of selective attention decoding at the 208–304 ms lag interval.

Pearson correlation between the behavioral speech score and the ρ_*Diff*_ revealed a significant correlation only between ρ_*Diff*_ and the speech score obtained with the CIS only listening mode (*r* = 0.712, *p* = 0.021) (Figure 8). A lack of significance in correlation between speech understanding performance and the selective attention correlation coefficients for AS only and CI+AS listening modes can be explained by the ceiling effect observed in the behavioral speech understanding performance.

## 4. Discussion

The main goal of this work was to investigate a possible electrophysiological measure of speech integration between electric and acoustic stimulation in bimodal CI users. An electrophysiological paradigm based on CAEPs to short stimuli showed the feasibility to record cortical responses with CIS only, AS only and CIS+AS listening modes. As an electrophysiological measure of speech integration, decoding of selective attention was proposed and validated in bimodal CI users. The results of the study confirmed that it is possible to decode selective attention in CI users despite the presence of CI electrical artifact in the EEG. Moreover, this work investigated how selective attention decoding is related to behavioral speech understanding performance. No bimodal benefit in speech understanding with respect to listening with the better ear was found, mainly due to the ceiling effects observed when listening with the CIS+AS and the AS only listening modes.

### 4.1. Speech understanding performance

Based on previous studies (e.g., Ching et al., 2004; Kong et al., 2005; Dorman et al., 2008; Potts et al., 2009; Vermeire and Van de Heyning, 2009; Yoon et al., 2015; Devocht et al., 2017), this work assumed a benefit in speech understanding for bimodal CI users when listening with CIS+AS listening mode in comparison to listening with the CIS only or with the AS only mode. Nevertheless, the interpretation of the reported results in the literature about the benefit of electric acoustic stimulation in bimodal CI users depends on the reference listening mode used to report the bimodal benefit and the inclusion criteria of the subjects participating in these studies. For instance, in agreement with the results of the current study, some previous studies have shown a benefit of bimodal listening compared to CIS only listening mode, but not compared to AS only listening mode (Mok et al., 2006; Devocht et al., 2017). In contrast, the study of Potts et al. (2009) observed a bimodal benefit compared to CIS only and to AS only, however, the authors of the study recruited candidates for bilateral CI implantation, i.e. with poor residual hearing.

Nevertheless, because of a ceiling effect observed in the speech scores with the CIS+AS and AS only listening modes, it was not possible to demonstrate a possible bimodal benefit compared to the best performing ear for some of the study participants. Despite this, two subjects obtained lower speech scores with the CIS+AS than with the AS only listening mode. Demographical data for these two subjects (Table 1) was analyzed and revealed long duration of deafness for subject 3, which can explain the reduction in speech understanding when listening with both sides compared to the better ear (Cohen and Svirsky, 2019). Moreover, a significant negative correlation between duration of deafness and speech scores with the CIS+AS listening mode was observed across all subjects (*r* = −0.846, *p* = 0.004). Subject 7 presented shorter duration deafness but reported not using the CI frequently in daily life, which probably explains the reduction in performance observed with the CIS+AS listening mode for this subject. To confirm the benefit of using the CI in daily life, subjects were additionally asked to answer questions regarding their listening experience in different acoustic situations. Results of the questionnaire are presented in the Supplementary material. Interestingly, all participants reported a benefit of using the CI in daily life. A number of previous studies showed benefit of bimodal hearing for CI users through improved quality of life (Galvin et al., 2019), drop in self-reported listening effort (Devocht et al., 2017) or even tinnitus suppression (Van de Heyning et al., 2008). Therefore, bimodal hearing provides benefits to CI users but these could not be measured through the speech understanding task proposed in this study.

Another possible explanation for the interference effect observed in two bimodal subjects when listening with CIS+AS is the reduced integration between electric and acoustic stimulation in our group of subjects. According to Yoon et al. (2015), bimodal benefit is greater in subjects that obtain similar performance with the CIS alone and the AS alone. Subjects recruited in the current study had normal or close to normal hearing with the AS only and relatively poor performance with the CIS only, due to the long duration of deafness prior to implantation. Therefore, this might have led to reduced integration between electric and acoustic stimulation. Moreover, Reiss et al. (2014) and Fowler et al. (2016) suggested that bimodal CI users can better integrate mismatched rather than matched spectral information across listening sides. The participants of the current study had a good residual hearing causing broad frequency range overlap across ears. As a result, this abnormal broad spectral integration may have lead to speech perception interference when listening with the CIS+AS compared to AS alone.

On the other hand, the interpretation of the results about bimodal benefit reported in the literature depends on the utilized materials and tests. The present work investigated the benefit of electric acoustic stimulation on speech understanding with a co-located target and interferer presented at the same level. The same speech material was presented in both ears, which does not allow to measure some binaural effects such as spatial release from masking or binaural squelch. Moreover, the interferer consisted of a speech signal which reduces speech understanding in CI users compared to the utilization of non-intelligible maskers, such as stationary or babble noise (Dieudonné and Francart, 2020). Studies using a similar paradigm as the one used in the current study observed no speech understanding improvement in bimodal CI users compared to the better ear performance (Vermeire and Van de Heyning, 2009; Galvin et al., 2019; Dieudonné and Francart, 2020). In the current study, we decided to use the same material for the behavioral and for the selective attention decoding paradigm such that the results of both experiments could be compared to each other. The selective attention paradigm, which has been extensively validated in our previous works in CI users, is based on a target and an interferer speech streams presented at the same level to reduce the effect of the CI artifact.

### 4.2. Cortical auditory evoked potentials

It was possible to measure CAEPs and to distinguish the N1P2 complex with all three listening modes for 9 out of 10 participants. Subject 1 was excluded from the analysis due to the low quality of the EEG signal caused by high impedances of the EEG electrodes. The peak-to-peak N1P2 latencies and amplitudes were in the range of 85–130 ms and 3–7 μV, respectively. These results are in agreement with the results reported in NH listeners (Martin et al., 2007; Stapells, 2009; Papesh et al., 2015) and CI users (Pelizzone et al., 1987; Ponton et al., 1996; Maurer et al., 2002; Sharma et al., 2002).

The highest N1P2 amplitude of 6.7 μV was obtained for the AS only condition. No significant difference between the N1P2 measured with the CIS+AS and the CIS only listening modes was observed. Previous studies have shown greater N1P2 responses with bilateral stimulation compared to monaural stimulation in NH listeners. The mentioned study claimed that the greater response evoked by the bilateral stimulus compared to the monaural stimulus can be explained by binaural integration or fusion of stimuli across both ears (Butler et al., 1969). In the current study higher N1P2 amplitudes for the CIS+AS listening mode compared to the CIS only or AS only listening modes were expected due to possible synergetic integration of electric and acoustic stimulation (Ching et al., 2001; Kong et al., 2005; Kong and Carlyon, 2007). However, the responses for the CIS+AS and the CIS only listening modes were not significantly different.

One possible explanation for the reduced bimodal response is the time processing difference or the lack of synchronization between the two listening sides. The time delay for the CIS is caused by the CI sound processor and the implant. The delay for an acoustic stimulus to reach the auditory nerve comprises ear canal, middle ear and the basilar membrane traveling wave delays. In this work, the processing delay for the CIS was measured through an oscilloscope and the delay for the AS was estimated from the literature. While the delay for the CIS is device dependent and has negligible variability across CI users with Oticon Medical CI, the time delay on the AS is less obvious, it depends on the individual anatomy and physiology of the ear, and it is frequency dependent due to the tonotopic organization of the cochlea. If to compare the estimated traveling wave delays provided by different authors utilizing different measurement techniques, a high variability across studies can be observed (Elberling et al., 2007). In this work, the delay between the two listening sides was not individually compensated, which may have caused reduced electric acoustic integration and consequently reduced bimodal CAEP responses. One possible solution for an individual delay compensation in bimodal CI users, is to correct the delay based on the wave V of the auditory brainstem response (ABR) as proposed by Zirn et al. (2015). However, the implementation of this procedure in the current work would have dramatically increased the time required to conduct the experiment. Nevertheless, the individual temporal synchronization between the two listening sides using ABRs has to be considered for future work.

### 4.3. Selective attention decoding

The topographical analysis of the decoder weight distribution for the forward model approach revealed weaker activation when listening with the CIS only than with the CIS+AS or the AS only listening modes. This weaker activation may be related to the difficulties experienced by the bimodal CI users to concentrate on the desired speech stream using the CIS only listening mode. The TRF morphology across lags for all three listening modes is consistent with previous reported results in NH listeners (Crosse et al., 2016) and CI users (Paul et al., 2020). For TRFs the highest N1P2 peak-to-peak amplitude was obtained when listening with the AS only, followed by the CIS+AS and the CIS only listening modes. The amplitude reduction of the TRF curve for CIS+AS listening mode may be explained by reduced integration between electric and acoustic stimulation or interference caused by the CI when listening with CIS+AS. Such an interference effect was observed at least in two subjects in the speech understanding performance test. Unfortunately, it was not possible to establish a relation between TRF amplitude and behavioral speech understanding performance in the current study due to ceiling effects observed in the speech understanding test with the AS only and CIS+AS listening modes. Moreover, a delay between TRF peaks for the AS only and the CIS only listening modes was observed. Therefore, the reduction of the TRF amplitude for the CIS+AS listening mode compared to the AS only listening mode can be also explained by the lack of the temporal synchronization between electric and acoustic stimulation.

As the TRF curve resembles the N1P2 complex of CAEPs, the individual N1P2 amplitudes from TRFs were compared to the N1P2 amplitudes of the CAEP responses.A significant correlation between both measures was observed for CIS only and AS only listening modes. For the CIS+AS listening mode no significant correlation was observed, possibly because a delay compensation between both sides was applied in the CAEP measurements but not in the selective attention paradigm. In the future, the impact of interaural delay on selective attention decoding should be further investigated.

The correlation coefficients of backward selective attention decoding with the CIS+AS and the CIS only listening modes obtained high values for the first lags, probably because of the contribution of residual CI artifact. As the target and interference were presented at 0 dB SIR, the contribution of the CI artifact is almost equal for both the attended and the unattended speech envelopes. Therefore, in absence of neural activity, the correlation coefficients to the attended and unattended envelopes should be almost identical, as demonstrated by an artifact model in our previous study (Nogueira et al., 2019a). In the current study, only a small difference between the attended and unattended correlation coefficients in the CIS only condition was observed. This is not surprising, taking into account the poor behavioral speech understanding performance obtained by the study participants when listening with the CIS only mode. Meanwhile, when listening with the CIS+AS mode, a significant difference between the attended and unattended correlation coefficients was observed. This result confirms the possibility to decode selective attention in CI users despite the presence of residual CI electrical artifact leaking into the EEG. Two peaks were observed at 100 and 220 ms. Coming back to the CI artifact model mentioned before, high correlation coefficients at early lags up to 80 ms followed by a decay ending at around 150 ms have been observed in our previous study (Nogueira et al., 2019a). Therefore, the first peak might be contaminated by the CI artifact, but the second peak might be less contaminated by the artifact. For this reason, this second peak could potentially be a valid parameter to compare selective attention decoding between different listening modes. The time occurrence of the second peak also corresponds to the late locus of attention reported by Power et al. (2012). Therefore, the lag interval of 208–304 ms was chosen for further analysis. Moreover, we focus our analysis on the difference between the attended and the unattended correlation coefficients, which further reduces the effect of the CI artifact as shown in previous studies (Paul et al., 2020; Nogueira and Dolhopiatenko, 2022). Besides that, the analysis of the difference between attended and unattended correlation coefficients might reduce the impact of some individual factors, such as artifact, skin thickness or electrode impedance. The comparison of the difference correlation coefficient revealed higher values for the CIS+AS and AS only listening modes compared to the CIS only listening mode, which is consistent with the speech understanding behavioral results. The correlation between selective attention decoding coefficients and behavioral data was significant only for the CIS only listening mode. A lack of significance for the CIS+AS and AS only modes can be explained by the ceiling effect in the speech understanding scores observed in these conditions. For this reason, the results of the current study cannot conclude whether selective attention decoding can be used as an electric acoustic speech integration measure. An extension of the dataset including bimodal CI users with less residual hearing or the use of different speech understanding performance tests to avoid ceiling or floor effects need to be considered for future work.

## 5. Conclusion

This work demonstrates that it is possible to decode selective attention in bimodal CI users. This result provides more evidence on the use of continuous EEG recordings to speech stimuli in CI users despite the presence of continuous electric artifact. The analysis of CAEPs and TRFs from selective attention decoding demonstrated an amplitude reduction when listening with CIS+AS relative to listening with AS only. The outcomes of this study may pave the way toward novel speech integration measures for bimodal CI users using EEG to continuous stimuli. However, further validation of these measurements are required.

## Data availability statement

The raw data supporting the conclusions of this article will be made available by the authors, without undue reservation.

## Ethics statement

The studies involving human participants were reviewed and approved by Ethics Committee of the Hannover Medical School. The patients/participants provided their written informed consent to participate in this study.

## Author contributions

HD implemented the algorithm and conducted the data collection and analysis. HD and WN contributed to the experimental setup and wrote the manuscript. WN conceived the study and contributed to data analysis and interpretation. All authors contributed to the article and approved the submitted version.
