# Corrigendum: Selective attention decoding in bimodal cochlear implant users

**DOI:** 10.3389/fnins.2023.1200637

**Published:** 2023-04-19

**Authors:** Hanna Dolhopiatenko, Waldo Nogueira

**Affiliations:** Department of Otolaryngology, Hannover Medical School and Cluster of Excellence Hearing4all, Hanover, Germany

**Keywords:** cochlear implant, selective attention, electric acoustic stimulation, electrophysiological measures, central integration, bimodal hearing, bimodal stimulation, electroencephalography

**Incorrect Funding**

In the published article, there was an error in the Funding statement. The Funding statement is not full. Original incorrect text: “This work was supported by the Deutsche Forschungsgemeinschaft (DFG, German Research Foundation) cluster of excellence EXC 2177/1. Part of these results is part of the project that received funding from the European Research Council (ERC) under the European Union's Horizon-ERC Program (Grant agreement READIHEAR No. 101044753-PI: WN).” The correct Funding statement appears below.

