# Supplementary material for: Selective attention decoding in bimodal cochlear implant users
Source: Front Neurosci. 2023 Jan 11;16:1057605. doi: 10.3389/fnins.2022.1057605 (PMC9874229; doi:10.3389/fnins.2022.1057605)
Supplement: Supplementary file 1 [file Data_Sheet_1.pdf]

Supplementary Material

0.1 Optimization of regularization parameter λ

The choice of the regularization parameter λ was based on the maximization of the peaks of TRF for the forward model and the maximization of the difference between the attended and the unattended correlation coefficients for the backward model. Figure S1 presents the TRFs and Figure S2 presents the correlation coefficients obtained using values of λ = [0.001, 0.01, 0.1, 1, 10, 100, 1000]. For the forward TRF, it can be observed that the highest amplitudes are obtained when λ = 100. For the backward model, the impact of λ on the correlation coefficients is less obvious. However, we can observe higher differences between the attended and the unattended correlation coefficients for the CIS only listening mode when using λ = 0.01.

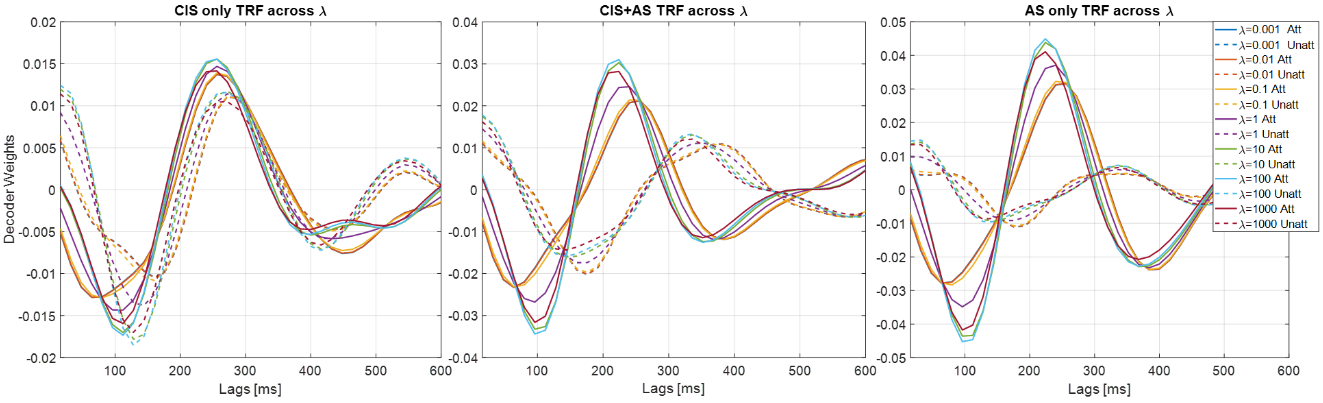

Figure S1. Attended and unattended temporal response functions (TRFs) across different λ values for CIS only (left), CIS+AS (center), AS only (right) listening modes.

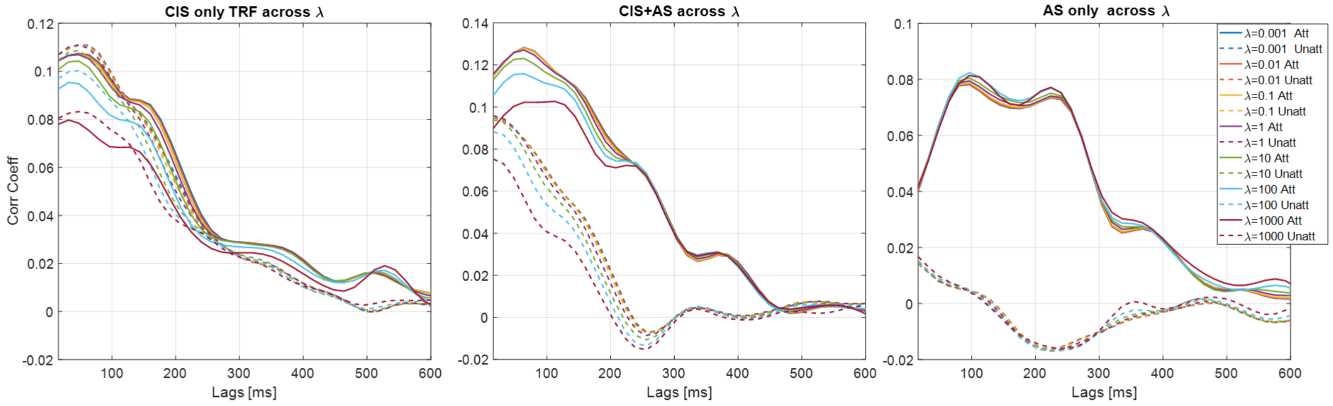

Figure S2. Attended and unattended correlation coefficients across different λ values for CIS only (left), CIS+AS (center), AS only (right) listening modes using the backward model for selective attention decoding.

0.2 Questionnaire on benefit of using the cochlear implant.

To confirm the benefit of using the CI in daily life for bimodal subjects, participants of the current study were asked to fill questionnaire. Eight questions were included in the questionnaire to assess the subjective CI benefit for different listening situations. The listening situations include the conversation to a person in quiet or in the presence of background noise, listening to TV, radio or music, ability to localize the sound source and estimate the loudness of a sound source. Participants were asked to compare their hearing abilities using the CI on a scale ranging from 1 ("Much easier without CI") to 10 ("Much easier with CI"). From Figure S3 it can be observed that all participant obtained a clear benefit of using the CI, especially in situations such as conversation with one person in quite or listening to TV or a radio speaker. The overall hearing performance with a CI obtained a score 8, which demonstrates their benefit of CI usage in daily life.

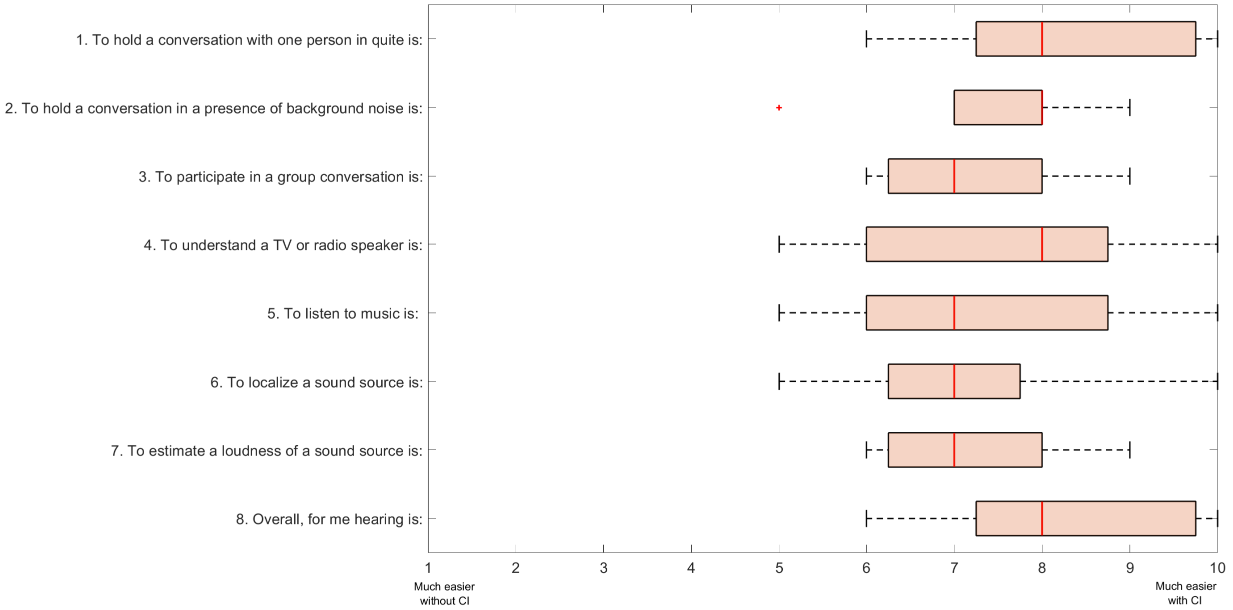

Figure S3. Questionnaire results on the benefits of using the cochlear implant in different listening situations for bimodal subjects participating in the current study.
